# Clinical effectiveness and costs of pre-hospital inhaled methoxyflurane for acute pain in trauma in adults: non-randomised control group study

**DOI:** 10.29045/14784726.2021.3.5.4.66

**Published:** 2021-03-01

**Authors:** A. Niroshan Siriwardena, Murray D. Smith, Elise Rowan, Rob Spaight

**Affiliations:** University of Lincoln; East Midlands Ambulance Service NHS Trust; University of Lincoln; University of Lincoln; East Midlands Ambulance Service NHS Trust

## Abstract

**Aims:**

Inhaled methoxyflurane, newly licensed in Europe for acute trauma pain in adults, has limited evidence of effectiveness in the pre-hospital setting. We aimed to investigate the clinical effectiveness and costs of methoxyflurane delivered when administered by ambulance staff compared with usual analgesic practice (UAP) for adults with trauma.

**Methods:**

A non-randomised control group design was used to compare methoxyflurane versus Entonox^®^ or parenteral analgesics. Verbal numerical pain scores (VNPS) were gathered over time in adults with moderate to severe trauma pain attended by ambulance staff trained in administering and supplied with methoxyflurane. Comparator VNPS were obtained from database records of UAP in similar patients. Clinical efficacy was tested using an Ordered Probit panel regression model of pain intensity linked by observational rules to VNPS. Scenario analyses were used to compare durations under analgesia spent in severe pain, and costs.

**Results:**

Over the 12-month evaluation period, 96 trained paramedics and technicians prepared 510 doses of methoxyflurane for administration to a grand total of 483 patients. Thirty-two patients reported side effects, 19 of whom discontinued early. Thirteen patients, 10 aged over 75 years, were nonadherent to instructions given on inhaler use. Modelling results included demonstration of statistically significant clinical effectiveness of methoxyflurane over each comparator (all p-values <0.001). Methoxyflurane’s time to achieve maximum pain relief was significantly faster (all p-values <0.001): 25.7 mins (95%CI 24.4–27.0) versus Entonox^®^ 44.4 (39.5–49.3); 25.8 (24.5–27.1) versus IV paracetamol 40.7 (34.6–46.9); 25.7 (24.4–27.0) versus IV morphine sulfate 41.9 (38.9–44.8). Scenario analyses of time spent in severe pain (VNPS on administration just scoring 10 reducing to a score of 7) were significantly less for methoxyflurane (all difference p-values <0.001): 7.6 mins (95%CI 6.5–8.7) versus Entonox^®^ 24.6 (20.1–29.0); 6.7 (5.6–7.7) versus IV paracetamol 23.0 (17.9–28.0); 6.9 (5.9–7.9) versus IV morphine sulfate 14.9 (13.3–16.6). Costing scenarios compared single-dose use of methoxyflurane versus Entonox^®^ and versus parenteral analgesics (80–20% weighted episode mix of morphine sulfate and paracetamol). In both scenarios, the benefits of methoxyflurane were achieved at higher cost: the additional cost per treated patient was £8.77 versus Entonox^®^ and £9.69 versus parenteral analgesics. The BNF list price for one vial containing a 3 mL dose of methoxyflurane for vaporisation in a Penthrox^®^ inhaler is £17.89 per pack.

**Conclusion:**

Methoxyflurane administered by ambulance clinicians reduced moderate or severe pain due to trauma in adults more rapidly compared to Entonox^®^ or parenteral drugs. Methoxyflurane provides a useful addition to pre-hospital analgesia.

